# Phase-based optoretinographic measurements of cones with a raster-scanning adaptive optics OCT are highly repeatable

**DOI:** 10.1364/BOE.591181

**Published:** 2026-04-22

**Authors:** Yao Cai, Maciej M. Bartuzel, Reddikumar Maddipatla, Robert J. Zawadzki, Ravi S. Jonnal

**Affiliations:** 1 Center for Human Ophthalmic Imaging Research (CHOIR), University of California, Davis Eye Center, Sacramento, CA 95817, USA; 2 Institute of Physics, Faculty of Physics, Astronomy and Informatics, Nicolaus Copernicus University, Torun, Poland; 3 Department of Biomedical Engineering, Wroclaw University of Science and Technology, Wybrzeze Wyspianskiego 27, 50-370 Wroclaw, Poland; 4 EyePod Small Animal Ocular Imaging Laboratory, Department of Cell Biology and Human Anatomy, University of California, Davis, CA 95616, USA

## Abstract

Optoretinography (ORG) is a class of methods that use noninvasive, all-optical techniques to measure stimulus-evoked responses in the retina. By conveying functional and structural information at once, it offers a valuable complement to existing functional assays such as visual acuity, visual fields, and electroretinography. Whether employed in basic scientific research or clinical testing, the utility of ORG depends on its sensitivity to changes in the cone response. This sensitivity depends in part on the intrinsic variation of photoreceptor responses. In ORG studies using adaptive optics (AO), single cone responses are sometimes reported, but more often the responses of many cones are pooled in a defined field of view (FOV) to convey an average cone response with improved signal-to-noise ratio (SNR). In this study, we quantify repeatability of cellular-resolution ORG measurements made with adaptive optics (AO) optical coherence tomography (OCT). First, we describe a model-fitting method for parameterizing and quantifying the ORG responses. Next, using model-derived parameters, we investigate two kinds of repeatability: among repeated measurements of individual cones and among repeated measurements of the pooled responses of cones in a 1° field of view. Repeated measurements of individual cones exhibited good consistency in the ORG amplitude. Although responses among neighboring cones can differ greatly, pooled responses of cones from the same field of view were highly repeatable for maximum outer segment elongation and the elongation rate. Both kinds of repeatability were assessed using the same data sets; test-retest pairs were acquired within 30-minute windows in an effort to isolate measurement noise from physiological changes that may be more appropriately considered signal. These findings demonstrate AO-OCT-ORG’s potential value in detecting changes in cone response due to experimental or therapeutic interventions, and its value in measuring longitudinal disease-related changes in response.

## 1. Introduction

Optoretinography (ORG) is an emerging functional imaging technique that uses light to probe the stimulus-evoked responses of retinal neurons, particularly photoreceptors. Combined with adaptive optics (AO), this technique may prove to be a noninvasive and objective way to measure subtle alterations in photoreceptor response at the cellular level, whether due to experimental manipulations, disease progression, or therapeutic intervention. It has the potential to provide an entirely new and complementary biomarker for retinal disease and recovery [1,2]. However, the clinical translation of ORG requires a comprehensive understanding of the method’s sensitivity to these alterations. That sensitivity, in turn, depends on the amount of uncertainty in ORG measurements, whether they are conducted on single cones or pooled responses of cones.

In this work we concern ourselves with two classes of measurement uncertainty: (1) variation in the response of a single cone over multiple repeated measurements, and (2) variation in the average behavior of cones from the same retinal location in a 1°x1° field of view (FOV). The latter of these depends on the variation among responses of neighboring cones, and we will describe that variation in the defined FOV as well. Both of these types of uncertainty affect ORG’s sensitivity to alterations in response. They are relevant for ORG approaches using scanning light ophthalmoscopy (SLO), whose quantitative methods depend on multiple measurements from the same cone [3] or parallel measurements from multiple cones [4]. Although the study involved three participants, it is not intended to address differences among subjects, as the number of subjects was insufficient to make any proper inferences.

Some noise sources contribute to both classes of uncertainty above: the effect of SNR-dependent phase noise [5], the effects of motion in the system or sample [6,7], and laser sweep noise [8]. Differing OCT-ORG implementations, including AO raster-point-scanning [9–13], AO line-scanning [14–16], hardware-based and computational AO enhanced full-field [17–20], and non-AO raster-scanning scanning [21–27] likely manifest these sources of noise differently, but the present findings may be of interest anyway, especially if these are not limiting sources of noise. Physiological changes, such as disc renewal [28,29], shedding [30], or dark/light adaptation may contribute additional uncertainty to repeated measurements of the single cone or mean responses over long periods. Therefore, to isolate baseline technical measurement repeatability from these physiological variables, the current study restricts the test-retest window to 30 minutes to establish the ORG measurement sensitivity of the system. Responses averaged among a sample of cones are also affected by sampling noise, which depends on the true variance of responses in the cones and the size of the sample. In each trial, the cones selected for analysis were determined by several factors: presence in the field of view, freedom from motion artifacts, sufficient sampling by A-scans, and 3D localization of their relevant features by the automated segmentation algorithm. Of course in in-vivo measurements, these factors vary randomly from trial to trial, and result in different subsets of cones being analyzed in each trial. In addition, even within small (1°) fields of view, topographical variation in anatomical parameters such as outer segment (OS) length, RPE-cone ratio, or health of the chorioretinal complex could contribute to inter-cone variability of response and thus impact the repeatability of pooled responses.

Although the outer segment response is likely tri-phasic, consisting of an initial rapid contraction followed by a larger elongation and then a slow late contraction toward baseline [31], in this study, we focus on the elongation. The elongation stage of the response has been hypothetically attributed to phototransduction-related osmotic swelling of the outer segment [15,32], hydration of the opsin proteins and the disc membrane they span [33], and/or mechanical force due to conformational changes in activated phototransductive enzymes [20]. To quantify this stage of the ORG response, we parameterized the response by fitting it with an overdamped oscillator model and extracting two parameters, the peak amplitude $\Delta {\mathrm{OPL}}_{\mathrm{fitting},\max}$ and the elongation rate $\tau_{a}$ , from the model fit. We also derived one figure of merit, the maximum outer segment elongation $\Delta {\mathrm{OPL}}_{\max}$ , from the data directly. The repeatability of these three parameters, both in single cone and pooled responses, is what we report below.

## 2. Methods

### 2.1. AO-OCT-ORG system

An earlier version of the AO swept-source (SS) OCT system used in this study is described in detail in a previous work [9]. It comprised an OCT system with a Fourier-domain mode-locked (FDML) laser (FDM-1060-750-4B-APC, OptoRes GmbH, Munich, Germany) operating at an A-scan rate of 1.64 MHz. The AO subsystem incorporated a pupil-conjugated deformable mirror (DM-97-15, ALPAO) and a custom-made Shack–Hartmann wavefront sensor [34]. The FDML laser power at the cornea was 1.8 mW. A superluminescent diode at 840 nm (Superlum Diodes Ltd, Cork, Ireland) served as the wavefront beacon, with power at the cornea of 150 µW. The system measured and corrected aberrations over a 6.75 mm pupil at a closed-loop rate of 10 Hz using custom AO control software [34,35], providing a theoretical lateral resolution of 3.2 µm in air based on the Rayleigh criterion.

A resonant scanner (SC-30, Electro-Optical Products Corp., Ridgewood, New York) oscillating at 5 kHz provided horizontal scanning, while a slower galvanometer scanner controlled vertical scanning. This raster scanning configuration, together with the FDML A-scan rate of 1.6 MHz, enabled a 28 Hz OCT volume rate over $1^{\circ }\times 1^{\circ }$ field of view. Each OCT volume consisted of 160 A-scans per B-scan and 172 B-scans, corresponding to sampling intervals of 1.875 µm and 1.744 µm in the fast and slow scanning dimensions. These fall short of the minimum sampling interval of 1.6 µm required by Nyquist-Shannon sampling theorem for the lateral resolution of 3.2 µm for continuous signal, a compromise we accepted in order to maintain a $1^{\circ }\times 1^{\circ }$ FOV.

A fiber-coupled LED (M565F3, Thorlabs, New Jersey) centered at 555 nm with 20 nm bandwidth served as the stimulus flash. We calculated the L and M cone bleaching levels using the cone spectral sensitivities provided by Stockman and Sharpe (2000) [36]. By measuring the LED spectral output with a spectroradiometer (PR670, Photo Research Inc., Syracuse, New York), we achieved a near-equal L/M cone quantal catch ratio of 1.06, ensuring that the stimulus bleached L and M cones at similar levels. The computation of photopigment bleaching is detailed in [23,37]. The stimulus beam diameter is 3.5 mm at the eye’s pupil plane, and 1.53° in diameter at retina to fully cover the 1° × 1° imaging FOV. A photopigment bleach level of 16 %, corresponding to 5.92×10^6^ photons/µm^2^ at the retina, was used in this study.

### 2.2. Imaging protocol

Subjects were dilated and cyclopleged using topical phenylephrine (2.5 %) and tropicamide (1.0 %). A bite bar and forehead rest were used to stabilize the eye during imaging. A calibrated LCD screen was used to display the fixation target. Before each recording, subjects underwent 5 minutes of dark adaptation by staying in a dark room with the eye to be imaged covered by an eye patch. Each recording was acquired at a volume rate of 28 Hz, with delivery of a 10 ms stimulus flash after the second volume. All experimental procedures complied with the Declaration of Helsinki and were approved by the Institutional Review Board of the University of California, Davis. Written informed consent was obtained from each participant after the study procedures and potential risks were fully explained. The combined illumination from the three sources met ANSI laser safety standard [38].

### 2.3. Subjects and imaging sessions

Three male subjects, aged 35-51 years, without known retinal disease, were tested. Each dataset consisted of three repeated trials, collected within 30 min. Moving away from the fovea, within a given FOV (e.g., 1°), the number of cones and the variation in their morphology (e.g., inner segment diameter, outer segment length) both fall. The eccentricity of 3° nasal 4° superior was selected to balance the number of tested cones with potential intrinsic variation in their shape and its potential effect on ORG response. Each ORG recording involved 42 volumes over 1.4 s following the onset of the flash. Our primary interest is in the repeatability of ORG measurements in the absence of intervening physiological changes. Many plausibly relevant physiological factors can change over time including diet, hydration, rest, circadian rhythms, and stress. To minimize the effects of these factors, repeatability of ORG responses was assessed by quantifying variability among the trials in a single session, separated by less than 30 min.

### 2.4. Data processing

**Fig. 1.**
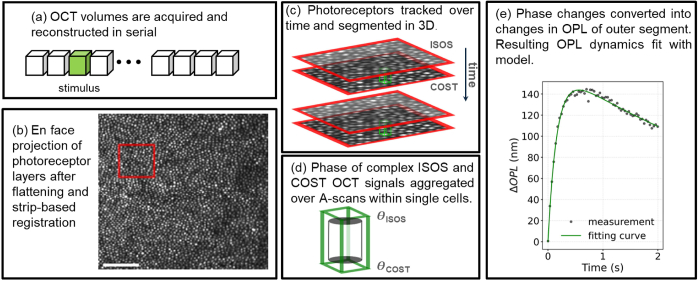
Key components of the ORG acquisition and processing pipeline. (a) Serially acquired AO-OCT volumes are reconstructed. Following flattening and strip-based registration, (b) *En face* projections of the cone mosaic are generated and used for photoreceptor localization. (c) Individual cones are tracked over time and segmented in 3D. (d) The phase difference between ISOS and COST layers is calculated for each cone. (e) The measurements of optical path length change over time are fit into an overdamped RLC model (Eq. (4)), as shown by the green curve.

Figure 1 illustrates the ORG data processing pipeline. OCT data were first reconstructed with numerical k -linearization and dispersion compensation. After numerically flattening the resulting volumes, the inner-outer segment junction (ISOS) and cone outer segment tip (COST) layers were automatically identified in each volume [29]. *En face* projections of the cone mosaic from each volume were then generated and used for strip-based registration [39] to correct lateral eye motion. Datasets containing blinks or saccades at the onset of the stimulus flash were excluded from further analysis. The registered *en face* projections were used for the identification and localization of individual photoreceptors. Each cone photoreceptor was tracked over time and segmented in 3D [29]. Finally, ISOS–COST phase differences were extracted for individual cones at each time point $t_{n}$ as below [9]:

(1) $${\left(\Delta \phi \right)}_{t}=∠\left(\frac{1}{m}\sum_{i=1}^{m}A_{i}\times B_{i}^{*}\right)$$

where A is the complex OCT signal measured at COST layer, $B^{*}$ is the complex conjugate of the OCT signal measured at ISOS, and m is the number of A-scans taken into account within each cone. In the present study, we segmented cones using a 7×7 pixel region. From this region containing the cone, the 9 brightest A-scans (m=9 ) were selected at each time point for ORG processing. After subtracting the initial phase (the average from the first two baseline measurements without stimulus flash), the change in optical path length ΔOPL at time t was calculated based on the imaging wavelength λ centered at 1063 nm and the unwrapped phase series $\Delta \Phi_{0}$ :

(2) $$\Delta OPL(t)=\frac{{\left(\Delta \phi_{0}\right)}_{t}}{4\pi }\lambda$$

To quantify the overall cone outer segment elongation, we proposed an overdamped RLC model [31] to fit the measured ORG signal as shown in Fig. 1(e). The measured optical path length change ( ΔOPL ) was expressed as:

(3) $$\Delta \mathrm{OPL}=\Delta {\mathrm{OPL}}_{\text{fit}}+\epsilon_{\text{residual}}$$

(4) $$\Delta {\mathrm{OPL}}_{\mathrm{fit}}=A_{1}\left(-e^{-t\tau_{a}}+e^{-t\tau_{b}}\right)$$

where $\Delta {\mathrm{OPL}}_{\text{fit}}$ is the model-fitted optical path length change, and $\epsilon_{\text{residual}}$ is a residual term that accounts for measurement noise and unmodeled physiological effects.

The root-mean-square (RMS) fitting error was calculated as:

(5) $${\mathrm{RMS}}_{\mathrm{fit}}=\sqrt{\frac{1}{N}\sum_{t=0}^{t_{N-1}}{\left(\Delta {\mathrm{OPL}}_{\mathrm{fit}}(t)-\Delta \mathrm{OPL}(t)\right)}^{2}}.$$

where $t_{N-1}$ is the terminal time point of the ORG recording sequence following the onset of the stimulus flash at t = 0 s, and N represents the total number of sampled time points. Cells were automatically filtered using an empirical RMS fitting error threshold (below 30 nm), retaining the majority of cones, with ∼70% to ∼90% included for analysis. The others were rejected due to poor fit, possibly due to registration or segmentation noise. The threshold of 30 nm is somewhat arbitrary, so we re-ran the analysis using thresholds of 20 nm and 40 nm as well. The resulting CoV values did not differ much in these three cases, and was ≤ 2.4% for all subjects at all thresholds. The lowest CoV values, ≤ 1.2% , resulted from the 30 nm threshold.

Three ORG-derived parameters were quantified from each fit to evaluate the overall ORG response: (1) $\Delta {\mathrm{OPL}}_{\max}$ (unit: nm): the maximum outer segment length elongation, calculated as the average of the five highest values of ΔOPL ; (2) $\Delta {\mathrm{OPL}}_{\mathrm{fitting},\max}$ (unit: nm): the peak amplitude derived from the RLC model fit (Eq. (4)) $\Delta {\mathrm{OPL}}_{\mathrm{fit}}$ ; (3) $\tau_{a}$ (unit: s^−1^): the parameter from the model (Eq. (4)) describing the rate of the OS elongation [31]. While a longer acquisition duration would be needed to estimate the late contraction rate ( $\tau_{b}$ ) accurately, our analysis focuses specifically on the peak OPL change ( $\Delta {\mathrm{OPL}}_{\mathrm{fitting},\max}$ ) and the initial elongation rate ( $\tau_{a}$ ), both of which manifest most strongly within the first 0.5 s post-stimulus. Thus, an acquisition time longer than 1.0 s is sufficient to capture these dynamics reliably.

ORG recording for 1.4 s is sufficient for estimation of the ORG amplitude parameters ( $\Delta {\mathrm{OPL}}_{\max}$ and $\Delta {\mathrm{OPL}}_{\mathrm{fitting},\max}$ ), since the peak amplitude is reached in <1 s. To determine whether $\tau_{a}$ was affected by the recording length, we extracted $\tau_{a}$ from model fits to truncated versions (1.0 s to 1.2 s) of the ORG recordings and found that they differed from the full 1.4 s recording by <0.4 % (see Supplement 1 Fig. S1). This suggests that prolonging the ORG recording would have minimal effect on estimates of $\tau_{a}$ .

## 3. Results

### 3.1. Single-cone test-retest reliability

**Fig. 2.**
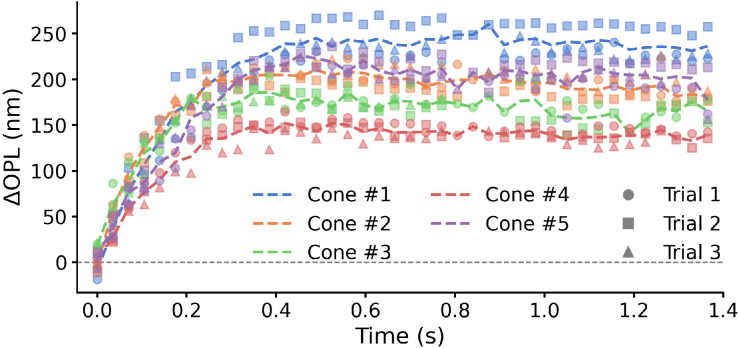
Representative ORG responses from five individual cones across three repeated trials from subject 1. Colors indicate individual cones, and different markers denote repeated trials. Dashed lines are averages of repeated measurements of the same cone. Missing data points are due to eye movements that transiently prevented their visualization and segmentation.

To assess test–retest reliability of single-cone ORG measurements, we analyzed ΔOPL from 280 cones co-registered across three trials in each of three subjects. Representative ORG traces from five cones are shown in Fig. 2. The data demonstrate high consistency across three repeated trials for each individual cone, in contrast to the pronounced inter-cone variability observed in response amplitude and kinetics (e.g., cone #1 exhibits a maximum OS elongation of approximately 250 nm, whereas cone #4 reaches only 150 nm).

We first computed three ORG parameters including the maximum outer segment length elongation $\Delta {\text{OPL}}_{\text{max}}$ directly from each ORG recording, as well as $\Delta {\text{OPL}}_{\text{fitting,max}}$ and $\tau_{a}$ , both derived from the RLC model fit.

Repeatability was evaluated using the coefficient of variation (CoV) and a two-way mixed-effects model intraclass correlation coefficient (ICC(2,1)) [40,41]. ICC values above 0.75 are said to indicate ’excellent’ repeatability [42]. For each subject, the overall CoV was computed as the root-mean-square (RMS) of the CoV across 280 cones.

As summarized in Table 1, the amplitude parameters ( $\Delta {\text{OPL}}_{\text{max}}$ and $\Delta {\text{OPL}}_{\text{fitting,max}}$ ) demonstrated high consistency. The CoV for these two parameters ranged from 5.5 % to 7.8 %, indicating low variability in repeated measurements and strong agreement between the experimental data and the model fit. Single-measure ICC values for these parameters ranged from 0.81 to 0.93, with the bounds of the 95 % confidence intervals (CIs) consistently exceeding the 0.75 threshold for excellent agreement, confirming good repeatability.

**Table 1.** Test-retest repeatability of single cone ORG responses: (1) Intraclass correlation coefficient (ICC) and 95 % confidence intervals (CIs) for maximum optical path length change ( $\Delta {\mathrm{OPL}}_{\max}$ , $\Delta {\mathrm{OPL}}_{\mathrm{fitting},\max}$ ) and elongation rate ( $\tau_{a}$ ); (2) Coefficient of variation (CoV, %) calculated as the ratio of the standard deviation to the mean.

Subject #	$\Delta {\mathrm{OPL}}_{\max}$		$\Delta {\mathrm{OPL}}_{\mathrm{fitting},\max}$		$\tau_{a}$	
		
	CoV (%)	ICC (95 % CI)	CoV (%)	ICC (95 % CI)	CoV (%)	ICC (95 % CI)
1	5.6	0.88 [0.85, 0.90]	5.5	0.86 [0.84, 0.89]	15.6	0.56 [0.50, 0.62]
2	5.8	0.82 [0.80, 0.84]	5.8	0.81 [0.77, 0.85]	12.7	0.53 [0.44, 0.61]
3	7.8	0.88 [0.85, 0.90]	6.3	0.93 [0.92, 0.94]	13.3	0.74 [0.69, 0.78]

In contrast, the elongation rate ( $\tau_{a}$ ) exhibited higher variation, with CoV values ranging from 12.7 % to 15.6 % for three subjects. The ICC values for $\tau_{a}$ were notably lower, ranging from 0.53 to 0.74 .

Because CoV depends on the standard deviation (rather than variance) of measurements, we chose to aggregate CoV values from multiple cones by computing the RMS of the cones’ CoV values, instead of averaging them. To validate this method we compared the aggregate value resulting from RMS to that resulting from averaging. Our analysis was restricted to 280 overlapped cones demonstrating ORG responses across all three trials. We found that the CoV average for $\Delta {\text{OPL}}_{\text{max}}$ (5.1%, 5.4%, and 6.8%) and $\tau_{a}$ (14.1%, 10.0%, and 11.7%) closely aligned with the CoV RMS (5.6%, 5.8%, 7.8% for $\Delta {\text{OPL}}_{\text{max}}$ ; 15.6%, 12.7%, 13.3% for $\tau_{a}$ ). Both the average and the RMS of CoVs exhibited identical trends across all three subjects. The minimal discrepancy between the simple average and the RMS computations confirms that our overall variance calculations (RMS CoV) are not artificially inflated by low-amplitude outliers.

These results indicated that while the magnitude of OS elongation was highly stable and reproducible in single-cone ORG measurement, the dynamics of the elongation process, represented by $\tau_{a}$ , were more sensitive to measurement noise and/or real physiological changes.

### 3.2. Test-retest repeatability of pooled cone responses

Pooling responses among cones has many potential benefits, including improvement of the response SNR and facilitation of concise descriptions of retinal health. As described above, a model-fitting framework was employed to parameterize individual cone responses. The resulting parameters were averaged among cones and the repeatability over three trials of these average parameters was quantified. The number of cones from which responses were aggregated varied between 400 and 900 among trials and subjects. As described above, for a given retinal location, the exact subset of sampled cones varied from trial to trial. Thus different samples were drawn from the 1-degree FOV population of cones, and used to compute the pooled responses. The three parameters used to quantify their responses were $\Delta {\mathrm{OPL}}_{\max}$ , $\Delta {\mathrm{OPL}}_{\mathrm{fitting},\max}$ , and $\tau_{a}$ .

**Fig. 3.**
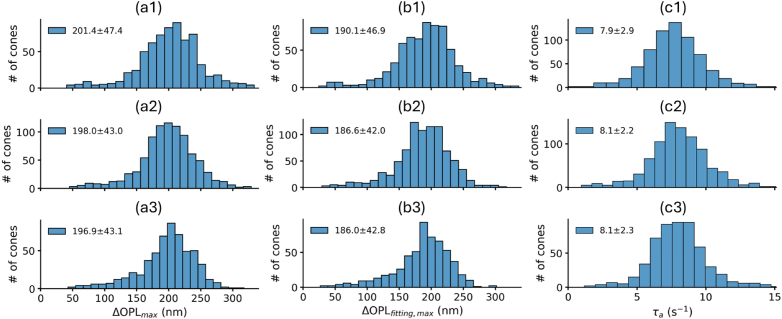
Histograms of three ORG parameters—(a) $\Delta {\mathrm{OPL}}_{\max}$ , (b) $\Delta {\mathrm{OPL}}_{\mathrm{fitting},\max}$ , and (c) $\tau_{a}$ —from three repeated trials (1–3) in Subject 2. Means ± standard deviations are indicated in each panel. Summaries of the results from all three subjects are given in Table 2.

For any given subject and parameter, the distribution among cells of the response parameter was qualitatively stable over the three trials. The distributions of all three response parameters over three trials from Subject 2 are shown in Fig. 3. Each panel’s legend depicts the distribution mean ± standard deviation. The mean value describes the averaged ORG response, and the standard deviation characterizes inter-cone variability across the FOV. Both mean values and standard deviations were qualitatively similar across trials.

**Table 2.** Summary of cone-pooled average of ORG parameters at 16 % bleach level over 1° FOV for three subjects. Each parameter is presented as mean ± standard deviation. Coefficient of variation (CoV, %) is computed as the ratio of the standard deviation to the mean.

Subject #	Δ ${\mathrm{OPL}}_{\max}$		Δ ${\mathrm{OPL}}_{\mathrm{fitting},\max}$		$\tau_{a}$	
		
	Average (nm)	CoV (%)	Average (nm)	CoV (%)	Average ( $s^{-1}$ )	CoV (%)
1	190.0 ± 1.7	0.9	183.9 ± 1.4	0.8	6.4 ± 0.11	1.7
2	198.8 ± 1.9	1.0	187.7 ± 1.8	1.0	8.0 ± 0.09	1.1
3	159.7 ± 1.4	0.9	146.2 ± 1.7	1.2	8.8 ± 0.07	0.8

When comparing the maximum OS elongation from direct measurement ( $\Delta {\mathrm{OPL}}_{\max}$ , Fig. 3(a)) with the maximum elongation derived from the fitting model ( $\Delta {\mathrm{OPL}}_{\mathrm{fitting},\max}$ , Fig. 3(b)), the mean values of the latter were consistently ∼ 10 nm lower than the former across all trials, while the standard deviations remained nearly identical. This suggests a bias introduced by selection of the five highest values of ΔOPL , and provides some confirmation that the proposed RLC model [31] reliably represents ORG responses. The distributions and descriptive statistics for elongation rate ( $\tau_{a}$ ) were also qualitatively stable across trials, as shown in Fig. 3(c) for Subject 2.

The repeatability of the ORG parameters across three repeated trials was quantified using the coefficient of variation (CoV). Table 2 summarizes the average values of the three studied ORG parameters, along with standard deviations of the means and CoV. The pooled average for both the ORG amplitudes ( $\Delta {\mathrm{OPL}}_{\max}$ , $\Delta {\mathrm{OPL}}_{\mathrm{fitting},\max}$ ) and the elongation rate ( $\tau_{a}$ ) demonstrated exceptional stability, with CoV values consistently below 2 %. Specifically, the CoV for the averaged maximum OS elongation length was approximately 1 %, demonstrating high reproducibility for repeated measurements. This performance is comparable to the CoV of 4.1 % previously reported for longitudinal measurements [43].

The test-retest repeatability for pooled responses is limited by several sources of variation or noise. Some of these are identical to sources of variation in retests of single cones: photon (phase) noise, laser noise, and segmentation noise. However, when pooling responses, two additional factors arise due to sampling differences among trials: the true dispersion of the response parameters among cells and the number of cells sampled. While the true dispersion of the parameters is not known, the dispersion of the sample distributions provides an estimate of it. Thus we computed the standard deviation of each parameter distribution and the stability of these standard deviations as well (Table 3). Across repeated trials, the CoV of these standard deviations ranged from 4.6 % to 8.6 % for $\Delta {\mathrm{OPL}}_{\max}$ and 7.7 % to 12 % for $\tau_{a}$ . These values indicate high stability of the distributions, and thus reliability of the pooled average responses.

**Table 3.** Repeatability of the statistical dispersion of ORG response parameters. The dispersion of ORG response parameters was quantified using standard deviation (STD). This value changed little among trials, with CoV ≤6.7% , ≤8.6% , and ≤12.0% for the three parameters.

Subject #	Δ ${\mathrm{OPL}}_{\max}$		Δ ${\mathrm{OPL}}_{\mathrm{fitting},\max}$		$\tau_{a}$	
		
	STD (nm)	CoV (%)	STD (nm)	CoV (%)	STD (s $s^{-1}$ )	CoV (%)
1	46.4 ± 3.0	6.4	48.9 ± 4.2	8.6	2.1 ± 0.20	9.6
2	44.5 ± 2.0	4.6	43.9 ± 2.2	4.9	2.5 ± 0.30	12.0
3	48.7 ± 3.3	6.7	48.0 ± 3.5	7.2	4.4 ± 0.34	7.7

The stability of the inter-cone standard deviation across trials, along with the high single-cone repeatability described above, suggests that the observed dispersion in cone responses is not stochastic noise, but rather due to inherent physiological diversity in individual photoreceptors. This could arise from many factors including baseline OS length and disc renewal/shedding, optical factors such as coupling efficiency of the stimulus light, and intrinsic variations in homeostatic support from surrounding Müller cells, RPE cells, and choroidal structure. While the stimulus light was designed to stimulate L-cones and M-cones equally, it is much less efficient at stimulating S-cones. The left side tails of distributions in Fig. 3 are consistent with the expected smaller responses of S-cones, and contribute to this diversity of response. To rule out contributions from the noise floor, we analyzed a no-stimulus control from Subject 3 ( Supplement 1 Fig. S2). System baseline optical path length fluctuations were approximately 15 nm peak-to-peak ( ∼ 2.5 nm RMS), confirming that the subset of cellular responses measuring 30 nm to 50 nm is not is not due to the phase noise in the ORG measurement.

Although the study was not designed to quantify inter-subject variability in ORG response, it is worth noting that we observed significant variability in ORG parameters among subjects. For example, Δ ${\mathrm{OPL}}_{\max}$ ranged from 159.7 nm for Subject 3 to 198.8 nm for Subject 2, showing approximately 24 % difference between the two subjects. Other studies have also shown significant inter-subject differences [23,31,44]. Further research is needed to better characterize the inter-subject differences in ORG responses.

## 4. Discussion

In this study, we demonstrated the high repeatability of our ORG measurement, but there were several limitations. A key limitation was its inability to quantify inter-subject differences in response. From the three subjects imaged, it appears that pronounced variability in response among subjects may exist. Quantification of this variability is an important first step in the design of cross-sectional or diagnostic ORG applications since it dictates the statistical power of such studies. The observed differences highlight the need for a normative ORG database to permit statistical inferences between subjects. An additional potential benefit of such a study would be the ability to make comparisons between test subjects and clinically and demographically similar norms. Factoring out effects of age, sex, cataract, intraocular lenses, and other variables would improve diagnostic sensitivity. We have begun edification of such a normative database, using the velocity-based protoclinical OCT system [23]. If a sufficiently significant cross-sectional hypothesis arises in our work, which can only be tested using AO-OCT-ORG, we would consider attempting to build a similar normative database using this method. Alternatively, we may try to harmonize the ORG results acquired with different methods and systems in such a way that norms from the former could be used to make cross-sectional inferences with AO-OCT-ORG data.

Another limitation of the study was our inability to determine the source of test-retest variation. Potential factors include SNR-dependent phase noise, slight differences in dark adaptation, homeostatic processes in the cones such as disc renewal and shedding, and positioning of the stimulus beam in the pupil (i.e., the Stiles-Crawford effect). Planned future work includes investigation of the effects of these parameters on ORG responses, using the AO-OCT-ORG.

Moreover, although the single cone and pooled average responses exhibit excellent repeatability, this was only demonstrated in three subjects. It is possible that these are not representative of the broader population of subjects, especially if we include subjects with diseases that affect photoreceptor function. Planned future work includes a similar test-retest repeatability assessment of the velocity-based method in subjects with and without retinal disease.

In this study we were constrained by the bandwidth of the system to study the repeatability of the elongation stage of the cone response. The repeatability of other components–the initial and late contraction stages–may be of interest too. We may try to investigate the former using our full-field AO-OCT system, capable of volume rates up to 1 kHz [45]. The latter poses additional challenges, as its estimation requires longer recording times, during which eye movements reduce the number of cones continuously imaged.

Lastly, because the imaging system is a scanning system and the stimulus is delivered with a single, un-scanned, full-field flash, there are inherent temporal offsets among the responses of different cones. This is a potential source of variation among estimates of $\tau_{a}$ and $\Delta {\mathrm{OPL}}_{max}$ . One way to address this problem would be to scan the stimulus light along with the imaging light, though we have not considered the technical challenges this would impose. Another possibility would be to interpolate all cone responses into a single time frame referenced to the stimulus onset, but this will introduce interpolation noise, the effect of which we do not know.

## 5. Conclusion

This study presents a characterization of single-cone and cone-pooled ORG test–retest reliability. We introduced a three-parameter model-fitting approach to describe optical path length changes at the single-cone level, which enables reliable characterization of ORG traces.

Using well-controlled stimuli and a carefully standardized image processing pipeline, we found excellent intra-cone test–retest reliability in Δ ${\mathrm{OPL}}_{\mathrm{fitting},\max}$ , with CoV around 6 % across three repeated measurements and single-measure ICC values above 0.8, while the elongation rate $\tau_{a}$ showed a higher variation.

Average responses within a 1° field of view demonstrated exceptional consistency across three repeated trials. Both the ORG amplitude and temporal dynamics yielded CoVs below 2 %, with the maximum optical path length change showing particularly robust at a CoV of approximately 1 % across all subjects. Furthermore, the inter-cone variation (standard deviation across the mosaic) for maximum OS elongation also remained stable (CoV: 5 %–9 %), exhibiting lower variability than the elongation rate $\tau_{a}$ , which showed a CoV of approximately 10 %.

Overall, our findings indicate that while single-cone measurements are subject to higher variability, the ORG amplitude is a significantly more reproducible parameter than the elongation rate ( $\tau_{a}$ ) for longitudinal assessment (see Table 1). By restricting our test–retest window to less than 30 minutes, we effectively isolated the system’s baseline sensitivity from long-term biological changes. The exceptional repeatability of cone-pooled measurements, particularly the averaged maximum OS elongation (see Table 2), highlights its potential as a robust and high-precision biomarker for distinguishing true physiological or disease-related changes.

## Supplemental information

Supplement 1 Supplementary Document (Source Files and Figures) https://doi.org/10.6084/m9.figshare.32005899

## Data Availability

Data underlying the results presented in this paper are not publicly available at this time but may be obtained from the authors upon reasonable request.

## Supplemental document

See Supplement 1 for supporting content.
