# Supplementary material for: Phase-based optoretinographic measurements of cones with a raster-scanning adaptive optics OCT are highly repeatable
Source: Biomed Opt Express. 2026 Apr 22;17(5):2598–609. doi: 10.1364/BOE.591181 (PMC13178630; doi:10.1364/BOE.591181)
Supplement: Supplementary file 1 [file boe-17-5-2598-s001.pdf]

Supplemental Document

Biomedical Optics EXPRESS

Phase-based optoretinographic measurements of cones with a raster-scanning adaptive optics OCT are highly repeatable: supplement

YAO CAI,[1,†,*] MACIEJ M. BARTUZEL,[1,2,3,†] REDDIKUMAR MADDIPATLA,[1,4] ROBERT J. ZAWADZKI,[1,4] AND RAVI S. JONNAL[1]

[1] *Center for Human Ophthalmic Imaging Research (CHOIR), University of California, Davis Eye Center, Sacramento, CA 95817, USA*
[2] *Institute of Physics, Faculty of Physics, Astronomy and Informatics, Nicolaus Copernicus University, Torun, Poland*
[3] *Department of Biomedical Engineering, Wroclaw University of Science and Technology, Wybrzeze Wyspianskiego 27, 50-370 Wroclaw, Poland*
[4] *EyePod Small Animal Ocular Imaging Laboratory, Department of Cell Biology and Human Anatomy, University of California, Davis, CA 95616, USA*
[†] *The authors contributed equally to this work.*
[*] *aocai@health.ucdavis.edu*

This supplement published with Optica Publishing Group on 22 April 2026 by The Authors under the terms of the Creative Commons Attribution 4.0 License in the format provided by the authors and unedited. Further distribution of this work must maintain attribution to the author(s) and the published article's title, journal citation, and DOI.

Supplement DOI: https://doi.org/10.6084/m9.figshare.32005899

Parent Article DOI: https://doi.org/10.1364/BOE.591181

Phase-based optoretinographic measurements of cones with a raster-scanning adaptive optics OCT are highly repeatable: supplemental document

To empirically verify that the estimation of the late recovery parameter τ_b does not "bleed" into the initial fast elongation rate (τ_a), we performed a step-wise truncation analysis on a representative dataset as shown in Figure S1. By decreasing the fitting time window from 1.4 sec down to 0.6 sec, we found that acquisition windows of 1.0 sec and above yield highly stable results for τ_a estimation, varying by less than 0.4% across all windows $\geq$ 1.0 s (τ_a ranges only from 8.24 s^{-1} to 8.29 s^{-1}). Because the fast elongation phase is unambiguously captured within the first 0.5 s, the algorithm tightly locks in τ_a. In contrast, the late recovery rate (τ_b) varies by 2% (0.150 s^{-1} to 0.153 s^{-1}) across these same windows. This confirms that late-stage τ_b estimation does not bleed into the extraction of the fast initial slope represented by τ_a. The varying τ_b merely applies a negligible baseline tilt that does not mathematically distort these early kinetics. Our 1.4 sec window is robust for estimating τ_a and $\Delta OPL_{fitting,max}$ by model fitting.

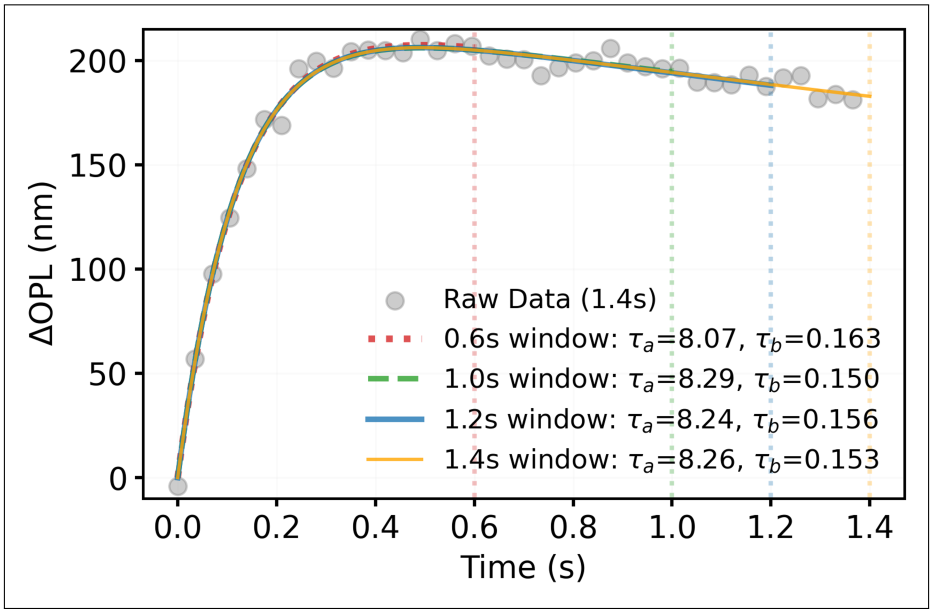

Fig. S1. Stability of the initial fast elongation rate (τ_a) estimation across 0.6 sec to 1.4 sec acquisition windows. While a 0.6 sec window is insufficient for optimal fitting, windows $\geq$ 1.0 s yield highly consistent τ_a estimates (8.24 s^{-1} to 8.29 s^{-1}, < 0.4% variation).

Figure S2 shows a continuous 1.5-second recording without a stimulus, phase noise causes baseline ΔOPL fluctuations of approximately 15 nm peak to peak (approx. 2.5 nm RMS), which represents the ORG measurement noise level by our AO-SS-OCT imaging system.

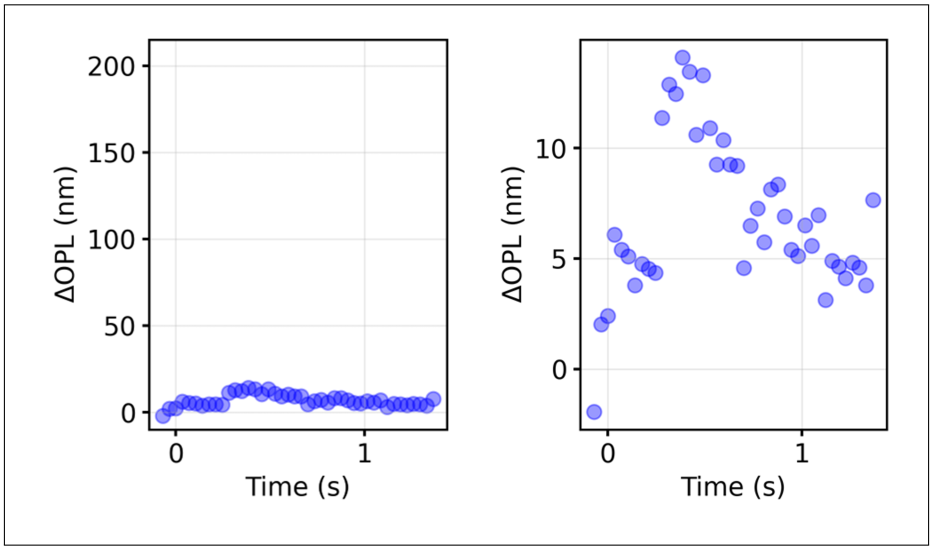

Fig. S2. No-stimulus ORG control measurement. Averaged optical path length change (ΔOPL) across 440 cells from Subject 3, recorded without a flash stimulus over 1.5 seconds (42 OCT volumes). The left panel displays the measurement on a typical ORG response scale under 16 % photopigment bleach level, and the right panel shows the magnified view. The phase noise floor is around 15 nm peak to peak in the ORG measurement by our AO-SS-OCT system.
